# Circulation collapse caused by intracardiac thrombosis associated with tranexamic acid administration: A case report

**DOI:** 10.1097/MD.0000000000027997

**Published:** 2021-11-24

**Authors:** Qing Xie, Chang-Jun Huang, Kai-Peng Gu, Yong-Xing Yao

**Affiliations:** Department of Anesthesia, First Affiliated Hospital, Zhejiang University School of Medicine, Hangzhou, P. R. China.

**Keywords:** circulation collapse, thrombosis, tranexamic acid, transesophageal echocardiography

## Abstract

**Rationale:**

Perioperative administration of tranexamic acid has been suggested to reduce bleeding and blood transfusion requirements in patients undergoing orthopedic surgery. Despite being sporadic, the potential risk for thrombotic complications cannot be ignored. However, intracardiac thrombosis associated with tranexamic acid administration is rare. We described a case of circulatory collapse caused by intracardiac thrombosis associated with tranexamic acid administration for a scheduled knee arthroplasty.

**Patient concerns:**

A 62-year-old male patient was scheduled for a right knee arthroplasty. He had a history of hypertension and had undergone surgery for treatment of right femur fracture 30 years previously. Other than a high platelet count (498 × 10^9^/L), results of laboratory investigations were within normal limits. The ultrasonic examination of both lower limbs showed no thrombosis. Upon sterilizing the surgical area, tranexamic acid (1.6 g) was intravenously administered after induction of anesthesia and intubation. Then the patient had a sudden circulatory collapse. Through cardiopulmonary resuscitation, the patient recovered spontaneous circulation. Transesophageal echocardiography revealed extensive thrombosis in the right atrium and ventricle.

**Diagnosis:**

Circulation collapse caused by intracardiac thrombosis

**Interventions:**

Thrombolytic therapy was recommended after urgent multidisciplinary consultation. Thus, urokinase was administered intravenously. Fifty minutes after thrombolysis, the mass in ventricle disappeared. A shrunken mass was observed in the right atrium. After another half an hour, no abnormal echoes were seen in the right heart chambers.

**Outcomes:**

The patient was discharged after 43 days without any organ dysfunction.

**Lessons:**

This case reminds clinicians that perioperative tranexamic acid administration may increase the risk of thrombosis, which needs focused attention from anesthesiologists. Prompt transesophageal echocardiography examination should be done to allow immediate diagnosis and effective thrombolysis therapy when unexplained cardiac arrest occurs during anesthesia.

## 1 Introduction

Perioperative administration of tranexamic acid (TXA) has been used to reduce bleeding and blood transfusion requirements in patients undergoing orthopedic surgery.^[1,2]^ Despite being sporadic, the potential risk for thrombotic complications cannot be ignored.^[3]^ However, intracardiac thrombosis associated with TXA administration is rare.^[4]^ Here, we describe a case of circulatory collapse caused by intracardiac thrombosis associated with TXA administration for a scheduled knee arthroplasty.

## 2 Case presentation

A 62-year-old male (height, 168 cm; weight, 90 kg) who complained of bilateral knee pain and limited mobility for 10 years was scheduled to undergo right knee arthroplasty. He had a history of hypertension, and was taking irbesartan hydrochlorothiazide 12.5 mg and levamlodipine besylate (5 mg daily). He had undergone a right femur fracture surgery 30 years previously. Systemic examination revealed no significant abnormalities. Other than a high platelet count (498 × 10^9^/L) laboratory investigations were within normal limits, with a prothrombin time of 10.9 s, activated partial thromboplastin time 24.4 seconds, and fibrinogen level of 3.38 g/L. Ultrasonic examination showed that left ventricular posterior wall thickness (diastolic) was 10 mm, left ventricular diameter (systolic) was 27 mm, and ejection fraction was 73%. No thrombosis or other abnormalities were seen in the blood vessels of either lower limb. Electrocardiogram and lung computed tomography also showed no abnormalities. The Caprini score was 3.

After electrocardiography, non-invasive blood pressure, and oxygen saturation monitoring, general anesthesia was induced with intravenous etomidate (18 mg), fentanyl (0.3 mg), and rocuronium (50 mg). After tracheal intubation, propofol (6 mg/kg/h) and remifentanil (0.2 μg/kg/min) were infused for maintenance of anesthesia.

Upon sterilizing the surgical area, TXA (1.6 g) was intravenously administered. Several minutes later, the patient's end-tidal carbon dioxide decreased from 35 to 7 mm Hg. Concomitantly, his blood pressure was 45/19 mm Hg, and oxygen saturation could not be analyzed. The patient developed ventricular fibrillation and severe acute pulmonary embolism was suspected. Epinephrine (100 μg followed by 1 mg) was administered immediately, and cardiopulmonary resuscitation was initiated. After electro-defibrillation, the patient recovered spontaneous circulation.

**Figure 1:**
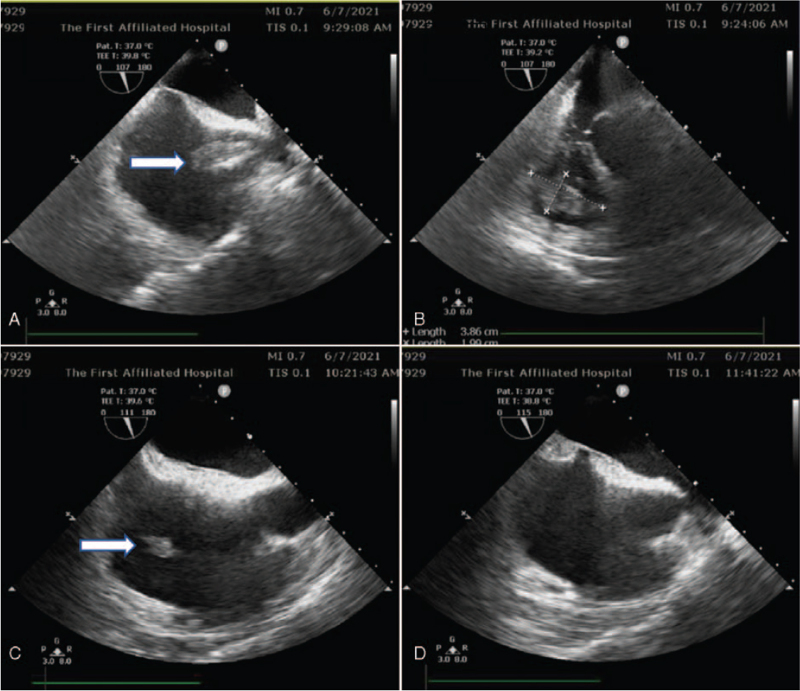
(A) A thrombus in the right atrium, which can be traced to the superior vena cava; (B) A thrombus in the right ventricle; (C) A shrunken mass was observed in the right atrium after fifty minutes thrombolysis; (D) The thrombus was not seen in the atrium.

The patient was catheterized in the radial artery and internal jugular vein for intensive monitoring. Hemodynamics was maintained with epinephrine (0.3 μg/kg/min). Blood gas analysis revealed a partial pressure of oxygen of 63.2 mmHg and partial pressure of carbon dioxide of 46.6 mmHg. Transesophageal echocardiography revealed a floating hypoechoic mass (5.9 × 1.8 cm) in the right atrium, which could be traced to the superior vena cava and right ventricle (3.9 × 2.0 cm), fluttering with heartbeat (Fig. 1A, B), while transthoracic echocardiography revealed no thrombosis in the jugular and femoral veins. After urgent multidisciplinary consultation, thrombolytic therapy was recommended. Thus, urokinase at 200,000 U for 10 minutes, followed by 300,000 U for 30 minutes was intravenously administered.

Fifty minutes after thrombolysis, the mass in the ventricle disappeared. Only a shrunken mass of 2.0 × 2.3 cm was observed in the right atrium (Fig. 1C). After another half an hour, no abnormal echoes were seen in the right heart chambers (Fig. 1D).

The operation was cancelled. After stable hemodynamics was preliminarily attained, the patient was transferred to the intensive care unit. Intraperitoneal hemorrhage and acute kidney failure were detected. After seven days of respiratory support and 11 days of renal replacement therapy, he returned to the general ward. He was discharged after 43 days without any organ dysfunction.

## 3 Discussion

In the present case, the patient experienced extensive intracardiac thrombosis and circulatory collapse associated with TXA administration before the operation. TXA has been widely used in clinical practice due to its antifibrinolytic effect. TXA has a high affinity with the lysine binding site of plasminogen, blocking the ability of fibrin to bind, which results in a decrease in fibrinolytic activity, thereby exerting a hemostatic effect.^[5]^ Studies have shown that TXA can reduce blood loss during the perioperative period in orthopedic surgery, without putting the patient at risk for thromboembolic events.^[6,7]^ However, there is still a potential risk for thrombotic complications. Myers et al suggested that TXA may be an independent risk factor for the formation of venous thrombosis in trauma patients.^[8]^ Furthermore, there have been reports of the development of left atrial thrombosis after intravenous TXA (1 g) administration.^[4]^ In our case, the patient had no apparent predisposing factors for thrombosis, and ultrasonic examination of blood vessels excluded preoperative deep venous thrombosis. The only risk factor he had was TXA administration after anesthesia. Combined with the quick dissolution of the thrombus, though other causes of hypercoagulability cannot be totally excluded, it is reasonable to infer that intracardiac thrombosis was associated with TXA infusion.

Intracardiac thrombosis is usually fatal. Acute thrombolytic therapy should be considered when a stable hemodynamics can be maintained.^[9]^ In the present case, the patient experienced extensive intracardiac thrombosis and circulatory collapse, although pulmonary embolism cannot be ruled out. This case alerts anesthesiologists of the possibility of TXA-associated thrombosis and the importance of transesophageal echocardiography examination for prompt diagnosis and treatment when unexplained circulatory collapse occurs during anesthesia.

## Acknowledgments

The authors would like to thank Editage (www.editage.com) for English language editing.

## Author contributions

**Conceptualization:** Yong-Xing Yao.

**Data curation:** Qing Xie.

**Writing – original draft:** Qing Xie and Chang-Jun Huang.

**Writing – review & editing:** Kai-Peng Gu and Yong-Xing Yao.
